# Comparative study of the impact of adjuvant trastuzumab and its biosimilars on cardiac function in HER2-positive early breast cancer patients: a single-center study

**DOI:** 10.3389/fonc.2025.1673372

**Published:** 2025-10-22

**Authors:** Lok Sze Joyce Au, Mai Yee Luk, Victor Ho Fun Lee, Kwok Keung Yuen

**Affiliations:** ^1^ Department of Clinical Oncology, Queen Mary Hospital, Hong Kong, Hong Kong SAR, China; ^2^ Department of Clinical Oncology, Li Ka Shing (LKS) Faculty of Medicine, The University of Hong Kong, Hong Kong, Hong Kong SAR, China

**Keywords:** trastuzumab, biosimilar, adjuvant, breast cancer, cardiac function, LVEF (left ventricular ejection fraction)

## Abstract

**Introduction:**

Two trastuzumab biosimilars have been introduced into the local public healthcare system since April 2021 as alternatives for treatment of human epidermal growth factor receptor (HER2) positive breast cancer. While their oncological efficacy compared to reference trastuzumab (Herceptin^®^) is well established, local comparative data on cardiotoxicity among the three formulations remain limited. This study evaluates the cardiac safety of Herzuma^®^ and Kanjinti^®^ compared with Herceptin^®^ in a single-center cohort.

**Methods:**

We conducted a retrospective review of 90 patients with HER2-positive early-stage breast cancer treated with adjuvant trastuzumab (Herceptin^®^, Herzuma^®^, or Kanjinti^®^) between January 2020 and January 2025 at Queen Mary Hospital, Hong Kong. Cardiac monitoring was performed via multi-gated acquisition (MUGA) scans every three months. Cardiotoxicity was defined as a ≥10% absolute decline in left ventricular ejection fraction (LVEF), LVEF <50%, or symptomatic heart failure. The primary study endpoint was time to cardiac event.

**Results:**

Cardiac events occurred in 19 patients (Herceptin^®^:18.6%, Herzuma^®^: 19.4%, Kanjinti^®^: 36.4%). No statistically significant differences in time to cardiac event were observed (log-rank p > 0.20). The odds ratio for events was highest in the Kanjinti^®^ group (vs. Herceptin^®^, OR = 2.50, p = 0.24). Median time to event was shortest in the Kanjinti^®^ group. No significant associations between cardiac events and cardiovascular risk factors identified.

**Conclusion:**

Trastuzumab biosimilars demonstrated comparable short-term cardiac safety to reference trastuzumab. While a higher event rate was observed in the Kanjinti^®^ group, the small sample size limits interpretation. Prospective studies with longer follow-up and larger patient number are warranted to better characterize long-term cardiac outcomes.

## 1 Introduction

Trastuzumab (Herceptin^®^) was first approved by the U.S. Food and Drug Administration (FDA) in 1998 for the treatment of human epidermal growth factor receptor (HER2) positive breast cancer (1). While its clinical efficacy is well established through landmark trials (2–4), the high cost of this monoclonal antibody has created challenges to healthcare sustainability, particularly in publicly funded health systems. To improve treatment accessibility while maintaining oncological outcome, trastuzumab biosimilars have been developed as alternatives over past years. Trastuzumab CT-P6 (Herzuma^®^) (5) and Trastuzumab ABP-980 (Kanjinti^®^) (6), both FDA-approved in 2018 and 2019, respectively, have demonstrated comparable safety and efficacy to Herceptin^®^ (7, 8). These biosimilars have been available at Queen Mary Hospital, Hong Kong since April 2021, as part of hospital policy to optimize resource allocation within the public healthcare system.

Cardiotoxicity is a well-recognized concern in trastuzumab-based therapy, as declines in left ventricular ejection fraction (LVEF) can lead to treatment modification or discontinuation, potentially compromising oncological outcomes. In the phase III CT-P6 equivalence trial, a ≥10% drop in LVEF was observed in 12.2% of patients receiving the biosimilar CT-P6 and in 11.5% of those receiving reference trastuzumab. Overall 1.8% in the CT-P6 group and 1.1% in the reference group experienced LVEF reduction ≥10% to below 50%, supporting cardiac safety equivalence. Separately, the LILAC trial evaluating ABP 980 reported LVEF decline of ≥10% and to below 50% in 2.8% of patients on the biosimilar versus 3.3% on the reference product, again demonstrated comparable safety. While these trials provide reassuring data, real-world evidence remains limited. Our study aims to evaluate the cardiac safety of trastuzumab biosimilars in a real-world setting by assessing treatment-related LVEF decline, providing practical insights into HER2-positive early breast cancer management, especially in balancing cost and toxicity considerations.

## 2 Materials and methods

This retrospective study was conducted in the Department of Clinical Oncology at Queen Mary Hospital, utilizing data extracted from the Clinical Management System and Electronic Patient Record system. A total of 90 patients with HER2-positive early-stage breast cancer who received adjuvant chemotherapy with Herceptin^®^, Kanjinti^®^, or Herzuma^®^ between January 2020 and January 2025 were included in the analysis. Patient characteristics such as age at trastuzumab initiation, cardiovascular risk factors including smoking history, hypertension, diabetes mellitus, hyperlipidemia, and history of coronary artery disease were retrieved. Tumor laterality, tumor and nodal staging by the 8th American Joint Committee on Cancer (AJCC) staging, were recorded.

Patients were treated with one of the two standard adjuvant chemotherapy regimens. The first regimen consisted of weekly paclitaxel (80 mg/m²) plus trastuzumab (loading dose 4 mg/kg, then 2 mg/kg) for 12 weeks, followed by trastuzumab (6 mg/kg) every three weeks for an additional 13 cycles (TH) (9). The second regimen comprised docetaxel (75 mg/m²) plus carboplatin (AUC = 6) plus trastuzumab (loading dose 8 mg/kg, followed by 6 mg/kg) every three weeks for 6 cycles, followed by trastuzumab every three weeks for 12 additional cycles (TTCH) (10). The brand of trastuzumab received, and exposure to radiotherapy and/or hormonal therapy, were documented.

Cardiac function was monitored using multi-gated acquisition (MUGA) scans at baseline and every three months throughout the treatment course, in accordance with departmental protocol and with reference to both product information (11) and the European Society for Medical Oncology (ESMO) guidelines (12). Collected data included baseline LVEF, intervals between MUGA scans, and changes in LVEF over time. Cardiac events were defined as an absolute LVEF decline of ≥10% from baseline, a reduction in LVEF to <50%, or the development of symptomatic heart failure. This definition was based on the European Society of Cardiology Position Statement (13), ESMO Clinical Practice Guidelines on cardiotoxicity (14) and the National Cancer Research Institute traffic light system for cardiac monitoring (15). These guidelines underpin our departmental protocol and were selected for their clinical relevance and applicability to local practice. Treatment modifications or discontinuations due to cardiac concerns were also documented.

Patients were excluded if they had distant metastases at diagnosis, incomplete clinical follow-up data related to cardiac outcomes, or had received more than one brand of trastuzumab during their treatment course. Those with prior or concurrent use of pertuzumab, prior exposure to anthracyclines, or those who did not complete trastuzumab treatment (except in cases of discontinuation due to a cardiac event) were also excluded. Patients who have not completed adjuvant trastuzumab by study end date were excluded.

Statistical analyses were performed to compare baseline characteristics and assess the association between cardiac events and trastuzumab treatment groups. The association between cardiac events and categorical baseline characteristics were analyzed using Chi-Square tests or Fisher’s Exact test where appropriate; Continuous variables were analyzed using analysis of variance (ANOVA) or Kruskal-Wallis test if data is not normally distributed. The time to cardiac event across treatment groups was analyzed using Cox proportional hazards model, and compared using the log-rank tests. A significance level of α ≤ 0.05 (two-sided) was considered statistically significant. All statistical analyses were performed using SPSS version 29.0 (IBM).

This research was approved by the Institutional Review Board of The University of Hong Kong/Hospital Authority Hong Kong West Cluster, Hong Kong (Ref No.: UW25-169). The requirement for patient consent was waived by the Committee due to the retrospective nature of the research.

**Figure 1:**
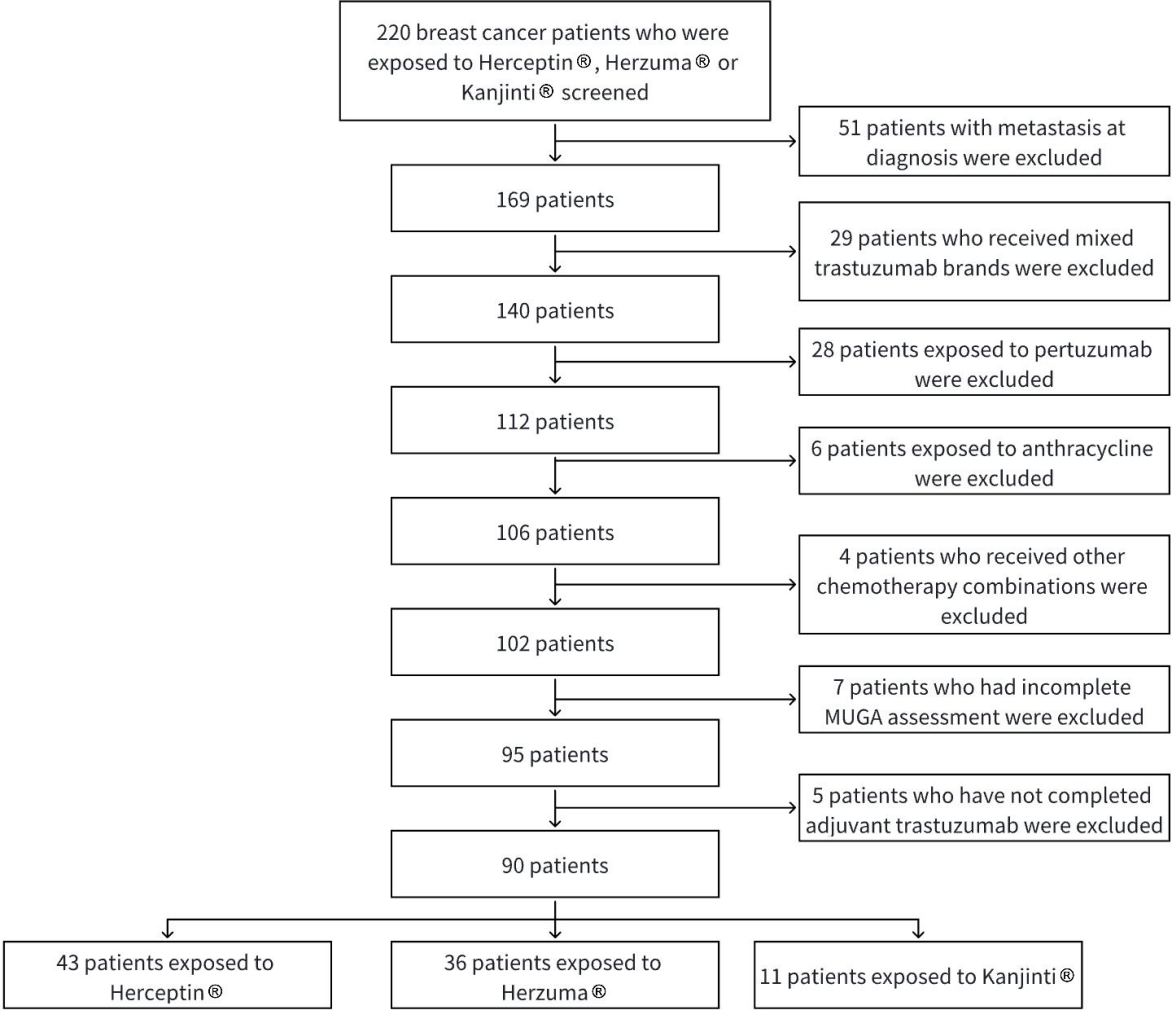
Study flowchart.

The selection of study subjects is illustrated in **Figure 1**.

## 3 Results

### 3.1 Patient characteristics

A total of 90 patients with HER2-positive early-stage breast cancer who received adjuvant trastuzumab were included in the analysis. Among them, 43 received Herceptin^®^, 36 received Herzuma^®^, and 11 received Kanjinti^®^. Baseline demographic and clinical characteristics were generally balanced across treatment groups (**Table 1**).

**Table 1:** Patient baseline characteristics.

Patient characteristics			Herceptin^®^ (n=43)	Herzuma^®^ (n=36)	Kanjinti^®^ (n=11)	P-value
Age (years, median [IQR])			56 (49.0, 61.5)	58 (52.0, 65.3)	62 (55.5, 68.5)	0.19
Cardiovascular Risk Factors	Ever Smoker		3 (7.0%)	1 (2.8%)	0	0.50
	Hypertension		12 (27.9%)	14 (38.9%)	5 (45.5%)	0.50
	Diabetes mellitus		6 (14.0%)	3 (8.3%)	4 (36.4%)	0.42
	Hyperlipidemia		7 (16.3%)	13 (36.1%)	1 (9.1%)	0.07
	History of coronary artery disease/Arrhythmia		1 (2.33%)	4 (11.1%)	0	0.16
Tumor characteristics	Left breast cancer		21 (48.8%)	16 (44.4%)	4 (36.4%)	0.75
	Right breast cancer		22 (51.2%)	20 (55.6%)	7 (63.6%)	0.75
	T stage	1 2 3 4	20 (46.5%) 21 (48.8%) 1 (2.3%) 1 (2.3%)	23 (63.9%) 13 (36.1%) 0 0	4 (36.4%) 7 (63.6%) 0 0	0.48
	N stage	0 1 2 3	26 (60.5%) 13 (30.2%) 1 (2.3%) 3 (7.0%)	29 (80.6%) 5 (13.9%) 2 (5.56%) 0	7 (63.6%) 4 (36.4%) 0 0	0.21
	Group Staging	IA IB IIA IIB IIIA IIIB IIIC	17 (39.5%) 0 9 (20.9%) 12 (27.9%) 1 (2.3%) 1 (2.3%) 3 (7.0%)	20 (55.6%) 0 11 (30.6%) 3 (8.3%) 2 (5.6%) 0 0	4 (36.4%) 0 3 (27.3%) 4 (36.4%) 0 0 0	0.10
Treatment characteristics	Baseline LVEF (%, mean ± SD)		71.3 ± 6.0	72.8 ± 5.5	68.6 ± 7.5	0.13
	MUGA interval (days, mean ± SD)		103.2 ± 28.9	110.4 ± 29.3	101.4 ± 28.1	<0.05
	Use with docetaxel-carboplatin		33 (76.7%)	29 (80.6%)	9 (81.8%)	0.89
	Use with paclitaxel		10 (23.3%)	7 (19.4%)	2 (18.1%)	0.76
	Adjuvant radiotherapy to left breast or chest wall		13 (30.2%)	8 (22.2%)	3 (27.3%)	0.72
	Adjuvant hormonal therapy		34 (79.1%)	27 (75.0%)	8 (22.2%)	0.87

The median age at trastuzumab initiation was comparable across the three groups (Herceptin^®^: 56 years, Herzuma^®^: 58 years, Kanjinti^®^: 62 years; p = 0.19). Cardiovascular risk factors, including hypertension, diabetes mellitus, hyperlipidaemia, and a history of cardiac disease, showed no significant differences between groups. Baseline LVEF, measured by MUGA scans, was 71.3% in the Herceptin^®^ group, 72.8% in the Herzuma^®^ group, and 68.6% in the Kanjinti^®^ group (p = 0.13). Similarly, tumor characteristics, including laterality and TNM staging (AJCC 8th edition), did not significantly differ among treatment groups (p = 0.10).

The Herzuma^®^ group had the longest mean MUGA monitoring interval at 110.4 ± 29.3 days, while the Kanjinti^®^ group had the shortest at 101.4 ± 28.1 days. A statistically significant difference in monitoring frequency was observed between groups (p <0.05). The shorter interval in the Kanjinti^®^ group is likely attributed to additional cardiac surveillance following an event, in accordance with the Traffic Light System guidelines established by the United Kingdom National Cancer Research Institute (NCRI) for managing trastuzumab-related cardiotoxicity. In the Kanjinti^®^ group, two patients underwent additional MUGA scans following notable LVEF declines: one patient had a repeat scan 35 days after an 18% drop in LVEF, and another had a scan 30 days after LVEF dropped from 58% to 44%. These episodes contributed to the overall shorter scan intervals observed in this group.

### 3.2 Cardiac events

A total of 19 cardiac events were recorded during the study period, with 8 events (18.6%) occurring in the Herceptin^®^ group, 7 (19.4%) in the Herzuma^®^ group, and 4 (36.4%) in the Kanjinti^®^ group. Comparisons of event rates between treatment groups showed that the odds of experiencing a cardiac event were similar between Herzuma^®^ and Herceptin^®^ (OR = 1.06, p = 1.00). While the Kanjinti^®^ group exhibited a numerically higher event rate, statistical analysis did not demonstrate a significant difference when compared to either Herceptin^®^ (OR = 2.50, p = 0.24) or Herzuma^®^ (OR = 2.37, p = 0.26). These findings suggest no statistically significant difference in cardiotoxicity risk among the three trastuzumab formulations, though the small sample size may have limited the power to detect a true difference (**Table 2**).

**Figure 2:**
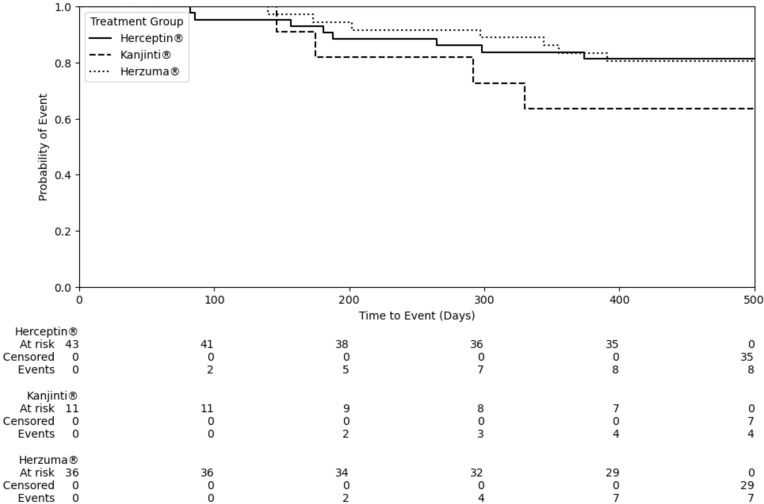
Time-to-cardiac event by treatment group.

The median time to a cardiac event was 265 days for Herceptin^®^, 297 days for Herzuma^®^, and 175 days for Kanjinti^®^. While no statistical significant difference was observed among the treatment groups (**Figure 2**; Log-rank test: Herceptin^®^ vs. Herzuma^®^, p = 0.98; Herceptin^®^ vs. Kanjinti^®^, p = 0.21; Herzuma^®^ vs. Kanjinti^®^, p = 0.21), the Kanjinti^®^ group exhibited a numerically shorter time to event. Notably, two patients in the Kanjinti^®^ group required treatment modifications due to cardiotoxicity: one patient temporarily suspended treatment, and another patient permanently discontinued her anticancer therapy after her LVEF dropped below 50%, and developed symptoms of congestive heart failure requiring medical therapy.

**Table 2:** Event rate among treatment groups.

Treatment Group	Event rate	Treatment Group	Odds ratio	P-value
Herceptin^®^	18.6%	Herzuma^®^ vs Herceptin^®^	1.06	1.00
Herzuma^®^	19.4%	Kanjinti^®^ vs Herceptin^®^	2.50	0.24
Kanjinti^®^	36.4%	Herzuma^®^ vs Kanjinti^®^	2.37	0.26

**Table 3** showed a summary of cardiac events in study population.

**Table 3:** Summary of cardiac events (*1: Drop in LVEF >=10%; 2: LVEF <50%; 3: Symptoms of congestive heart failure).

Treatment Group	Event*	Age	Time to event (days)	Baseline LVEF (%)	Maximum drop in LVEF (%)	Medical history	Radiotherapy to left breast/chest wall	Treatment suspension/discontinuation
Herceptin^®^ (n=8)	1	51	265	69	12	No	No	No
	1	56	181	73	10	No	Yes	No
	1	58	157	80	13	No	Yes	No
	1	66	82	79	11	Hypertension, Diabetes mellitus	No	No
	1	65	86	72	10	Hyperlipidaemia	No	No
	1	59	188	74	13	Minor coronary artery disease	No	No
	1	44	374	67	10	No	No	No
	1	54	298	72	12	No	Yes	No
Herzuma^®^ (n=7)	1	54	297	84	15	Hyperlipidaemia	No	No
	1	41	344	72	11	No	No	No
	1	56	355	79	11	Hypertension	Yes	No
	1	57	140	74	11	No	No	No
	1	56	173	80	10	No	No	No
	1	62	202	76	14	No	Yes	No
	1	43	391	74	10	No	No	No
Kanjinti^®^ (n=4)	1	28	146	77	18	No	Yes	Suspended 1 month
	1	64	175	72	10	No	No	No
	2,3	75	330	53	9	Diabetes mellitus	No	Discontinued
	1	74	292	74	12	Hypertension, Diabetes mellitus	Yes	No

### 3.3 Correlation between event and patient characteristics

No significant associations were observed between cardiac events and key cardiovascular risk factors, including history of coronary artery disease/arrhythmia (p = 0.62), diabetes mellitus (p = 1.00), hyperlipidaemia (p = 0.24), and prior left-sided breast/chest wall radiotherapy (p = 0.40). Similarly, no significant correlation was found between cardiac events and patient age ≥60 years (p = 0.89), smoking status (p = 1.00), or use of hormonal therapy (p = 0.97). Hypertension was the only characteristic that approached statistical significance (p = 0.10), suggesting a possible trend but not reaching the predefined threshold for significance. Additionally, choice of adjuvant chemotherapy (TTCH vs. TH) was not associated with an increased cardiac risk (p = 1.00). These findings indicate that within the current cohort, traditional cardiovascular risk factors did not appear to independently predict the likelihood of a cardiac event.

## 4 Discussion

### 4.1 Comparable cardiac safety of trastuzumab biosimilars in real-world use

HER2-directed therapy has become an integral component of managing HER2-positive breast cancer. A number of approved biosimilars of trastuzumab have become widely available in the past decade, providing a more affordable and comparably effective option to patients who might have financial difficulty of receiving brand-name trastuzumab. However, real-world data on cardiotoxicity of these biosimilars were not widely reported. This retrospective study assessed the cardiac safety of two trastuzumab biosimilars compared to the reference product trastuzumab in the adjuvant treatment of HER2-positive early breast cancer. Among the 90 patients included in this cohort, 19 experienced cardiac events. No statistically significant difference in event rate was found. Our findings are consistent with prior clinical trials and systematic reviews demonstrating that biosimilars exhibit comparable cardiac safety profiles to the reference trastuzumab (16, 17). Although the Kanjinti^®^ group had a numerically higher event rate (36.4%) and two instances of treatment modification or discontinuation, the small sample size of this subgroup (n=11) substantially limits the statistical power to draw firm conclusions. Furthermore, neither univariate nor multivariable analyses identified statistically significant associations between cardiac events and conventional cardiovascular risk factors. These findings suggest that baseline comorbidities alone may not fully account for individual susceptibility to trastuzumab-related cardiotoxicity, or that the study may have been underpowered to detect such relationships due to small sample size.

### 4.2 Cardiotoxicity surveillance tools: current tools vs emerging strategies

In this study, cardiotoxicity was assessed primarily by evaluating declines in LVEF and symptomatic heart failure, which remain the most widely used monitoring parameters in local oncological practice. These metrics are practical and more accessible, especially in resource-limited public healthcare settings. Compared to landmark clinical trials such as HERA (4) and NSABP B-31 (2), our study observed higher rates of cardiotoxicity, which may be attributed to variability in definitions of cardiac events across different guidelines and countries (18–20). Moreover, other cardiac manifestations such as arrhythmias, myocardial infarction, and myocarditis were not evaluated. The omission of these events, coupled with reliance on LVEF alone, may limit the sensitivity of our analysis in detecting subclinical or early-stage cardiotoxicity.

Emerging evidence suggests that a multimodal approach of integrating circulating biomarkers and advanced imaging techniques, which can enhance the early detection of trastuzumab-related cardiotoxicity. Several studies have demonstrated that biomarkers such as N-terminal pro–B-type natriuretic peptide (NT-proBNP), high-sensitivity troponin, Creatine kinase–MB isoenzyme (CK-MB), and Micro-ribonucleic acids (microRNAs) can identify myocardial injury prior to detectable LVEF reduction. For instance, Pillai et al. showed that microRNA-based panels could detect cardiotoxic changes even in asymptomatic HER2-positive breast cancer patients (21). Sawaya et al. and Díaz-Antón et al. further supported the utility of combining echocardiographic strain imaging with biomarkers to predict cardiac dysfunction with greater accuracy (22, 23). A systematic review by Thavendiranathan et al. reinforced these findings, highlighting the prognostic value of myocardial strain imaging in cardiac oncology (24). While promising, these tools are not routinely implemented in local settings due to cost and limited availability. Future prospective studies should consider incorporating these novel modalities to optimize cardiac monitoring in patients undergoing HER2-targeted therapy.

### 4.3 Economic evaluations and local implications

Internationally, several economic evaluations have demonstrated the cost-saving potential of trastuzumab biosimilars. For instance, a Taiwanese cost-effectiveness analysis showed that a trastuzumab biosimilar (not explicitly named in the study) combined with docetaxel was a cost-effective alternative to the reference trastuzumab in metastatic HER2-positive breast cancer, reporting an incremental cost-effectiveness ratio of NTD 811,050 (~USD $28,966) per quality-adjusted life year (QALY) gained (25). Similarly, a budget impact analysis across twenty-eight European countries estimated that using the trastuzumab biosimilar CT-P6 for breast and gastric cancers could lead to cumulative healthcare savings of €0.91 to €2.27 billion over a 5-year period (26).

While these findings explored the economic potential of biosimilars, they originate from healthcare systems with very different drug pricing structures, institutional policies, and patient demographics compared to Hong Kong. In our local public healthcare system, the adoption of trastuzumab biosimilars is still relatively new. As a result, data to support comprehensive cost-effectiveness evaluations, including QALYs and direct cost comparisons with the reference product, remain limited. Moving forward, research in the Hong Kong context should aim to assess cost-effectiveness of biosimilars in local population. This would involve incorporating local drug prices, treatment durations, and patient utility measures to better inform policy decisions and procurement strategies.

### 4.4 Study limitations and generalizability

This study has several important limitations. Its retrospective design introduces inherent selection bias, as treatment group allocation was determined by institutional policies and/or funding availability rather than randomization. As the two biosimilars were newly introduced, the number of patients is relatively small. The small sample size, particularly in the Kanjinti^®^ group, limits the generalizability and robustness of subgroup comparisons. Besides, the study’s follow-up duration was unable to capture the late-onset cardiotoxic effects, which were previously reported (27, 28). In addition, our results may not be generalizable in the modern era of neoadjuvant HER2-directed therapy before definitive surgery and more widespread use of dual HER2-targeted therapy with pertuzumab and trastuzumab in high-risk patients. Furthermore, we were unable to explore if modification or even early permanent discontinuation of treatment regimen will impact on survival as one recent Japanese study revealed that early discontinuation of trastuzumab treatment resulted in an increased mortality rate secondary to early disease relapse (29).

Future research should prioritize prospective study designs with larger, balanced cohorts, multicenter participation and extended follow-up periods to better evaluate the long-term cardiac safety of biosimilars in various clinical settings.

## 5 Conclusion

This study provides real-world data demonstrating that trastuzumab biosimilars (Herzuma^®^ and Kanjinti^®^) exhibit comparable short-term cardiac safety to reference trastuzumab (Herceptin^®^) in local clinical practice. While no significant differences in cardiac event rates were observed, variations in monitoring intensity and treatment modifications highlight the importance of ongoing cardiac surveillance and adherence to standardized monitoring protocols. Future studies should adopt prospective designs with larger, well-balanced cohorts and longer follow-up to clarify long-term cardiotoxicity risks and support evidence-based use of biosimilars.
